# Single Administration of AAV‐m*Atp6v1b2* Gene Therapy Rescues Hearing and Vestibular Disorders Caused by *Atp6v1b2*‐Induced Lysosomal Dysfunction in Hair Cells

**DOI:** 10.1002/advs.202408878

**Published:** 2025-03-11

**Authors:** Gege Wei, Shiwei Qiu, Xue Gao, Lu Zheng, Yijin Chen, Ying Ma, Haifeng Feng, Jinyuan Yang, Guojie Dong, Huiyi Nie, Weihao Zhao, Xiaoge Li, Guangqin Wang, Wei Xiong, Pu Dai, Yongyi Yuan

**Affiliations:** ^1^ Senior Department of Otolaryngology Head and Neck Surgery the 6th Medical Center of Chinese PLA General Hospital Chinese PLA Medical School Beijing 100853 China; ^2^ State Key Laboratory of Hearing and Balance Science Beijing 100853 China; ^3^ National Clinical Research Center for Otolaryngologic Diseases Beijing 100853 China; ^4^ Key Laboratory of Hearing Science Ministry of Education Beijing 100853 China; ^5^ Beijing Key Laboratory of Hearing Impairment Prevention and Treatment Beijing 100853 China; ^6^ Chinese Institute for Brain Research Beijing 102206 China; ^7^ Chinese Academy of Medical Science & Peking Union Medical College Beijing 100730 China; ^8^ School of Life Sciences Tsinghua University Beijing 100084 China; ^9^ Department of Otolaryngology PLA Rocket Force Characteristic Medical Center 16# XinWai Da Jie Beijing 100088 China; ^10^ Simp‐Gen Therapeutics Co., Ltd Suzhou 215425 China

**Keywords:** adeno‐associated virus (AAV), *Atp6v1b2*, cochlear hair cells, gene replacement, hearing loss, lysosome, vestibular hair cells

## Abstract

Haploinsufficiency of the ATP6V1B2, a subunit of V‐ATPases, underlies genetic disorders including Dominant deafness‐onychodystrophy (DDOD), deafness, onychodystrophy, osteodystrophy, mental retardation and seizures (DOORS), and Zimmermann‐Laband syndromes, all characterized by congenital hearing loss and onychodystrophy. Effective therapies for *ATP6V1B2*‐associated hearing loss remain elusive. The study generates a hair cell‐specific knockout mouse (*Atp6v1b2^fl/fl^*;*Atoh1^Cre/+^*) recapitulating the human phenotypes, with pathological features including hair cell loss and abnormal lysosomal morphology and function. To enhance therapeutic precision and minimize toxicity, an optimized adeno‐associated virus‐inner ear vector incorporating promoter enhancer 3 (AAV‐ie‐Eh3) is engineered. A single administration of AAV‐ie‐Eh3‐m*Atp6v1b2* into the scala media at postnatal days 0–2, prevented hair cell degeneration, restored lysosome morphology, and robustly rescued auditory and vestibular function for at least 24 weeks. The findings highlight the critical role of *Atp6v1b2* in lysosomal function and demonstrate AAV‐ie‐Eh3 as a potent gene delivery tool for inner ear therapy. This study establishes a novel therapeutic paradigm for *ATP6V1B2‐*associated hearing loss and vestibular dysfunction, with significant clinical potential.

## Graphical abstract

Wei et al. establish a hair cell‐specific conditional knockout mouse model (*Atp6v1b2^fl/fl^;Atoh1^Cre/+^*), and demonstrate the importance of *Atp6v1b2* for hair cell through maintaining the survival of lysosomes. A single administration of AAV‐ie‐Eh3‐m*Atp6v1b2* through scala media at P0‐P2 realizes function compensation and restores hearing and balance functions in *Atp6v1b2^fl/fl^;Atoh1^Cre/+^* animals.

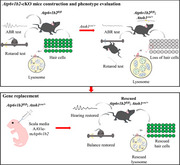

## 1 Introduction

Lysosomes serve as critical regulators of autophagy and play an indispensable role in maintaining cellular homeostasis. Autophagy dysfunction is a well‐established molecular mechanism underlying numerous neurodegenerative disorders, including Parkinson's disease, Alzheimer's disease, and lysosomal storage disorders.^[^ ^1^ ^]^ Extensive research on auditory hair cells (HCs) has demonstrated that lysosomal abnormalities contribute to various forms of hearing loss, including age‐related,^[^ ^2^ ^]^ noise‐induced,^[^ ^3^ ^]^ and drug‐induced hearing loss.^[^ ^4^ ^]^

ATP6V1B2, an vital subunit of vacuolar H^+^‐ATPase (V‐ATPase), functions as an ATP‐dependent proton pump essential for lysosomal acidification.^[^ ^5^ ^]^ Clinically, variants in the *ATP6V1B2* gene are associated with several syndromes, including DDOD (Dominant deafness‐onychodystrophy),^[^ ^6^ ^]^ DOORS (deafness, onychodystrophy, osteodystrophy, mental retardation and seizures) syndrome,^[^ ^7^ ^]^ and Zimmermann‐Laband syndrome (ZLS, gingival hypertrophy, coarse facial features, hypoplasia or aplasia of nails and terminal phalanges, intellectual disability, and hypertrichosis).^[^ ^8^ ^]^ These syndromes are characterized by congenital severe to profound sensorineural deafness, onychodystrophy, intellectual disability, and epilepsy.^[^ ^9^ ^]^ Over the last decade, researches from our group and others have elucidated the molecular variants, functions, and interactions of ATP6V1B2, revealing that its haploinsufficiency leads to reduced V‐ATPase hydrolysis and impaired hydrogen ion transport into lysosomes, resulting in elevated lysosome pH.^[^ ^6^, ^10^ ^]^ We have also generated a knockin mouse model carrying a frequent human *ATP6V1B2* variant, c.1516 C>T, which exhibits seizures and cognitive impairment.^[^ ^10a^ ^]^ Auditory brainstem response (ABR) tests in this model revealed hidden hearing loss by 3 months, which worsened by 7 months, differing from the human hearing phenotype.^[^ ^11^ ^]^ Another independently generated knockin *Atp6v1b2* ^emR506*^ mouse model displayed seizures and hyperactivity but lacked significant hearing loss at 14 weeks,^[^ ^12^ ^]^ partially corroborating our findings. Collectively, these knockin models fail to fully recapitulate the human deafness phenotype.

Although RNAscope analysis has shown that *Atp6v1b1* compensates for the loss of *Atp6v1b2* in hair cells, direct evidence supporting the functional significance of *Atp6v1b2* in hair cells remains limited. The fact that hearing loss in patients with *ATP6V1B2* defects can be mitigated by cochlear implants suggests that the gene's function is closely related to hair cells activity. To date, no effective molecular interventions or treatments have been developed.

Advances in gene therapy have established adeno‐associated virus (AAV) as a versatile delivery tool for treating inherited disorders.^[^ ^13^ ^]^ Their proven safety profile in both animal models and humans has made them a cornerstone of gene therapy for hearing loss.^[^ ^14^ ^]^ Various AAV serotypes exhibit distinct tropisms, enabling targeted delivery to specific cell types.^[^ ^15^ ^]^ AAV‐inner ear (AAV‐ie) has shown promise in targeting supporting cells and hair cells in the inner ear, making it a suitable candidate for inner ear gene delivery.^[^ ^16^ ^]^ However, the development of inner ear‐specific AAV gene therapies faces challenges, such as limited hair cell specificity and the durability of transgene expression.

In this study, to investigate the role of the *ATP6V1B2* gene in hearing, we generated a conditional knockout (KO) mouse model specifically targeting the *Atp6v1b2* gene in the inner ear hair cells. This model accurately recapitulates the auditory phenotype observed in clinical patients. After screening five AAV serotypes in vivo and comparing the conventional CAG promoter with enhancer 3, we identified AAV‐ie‐Eh3 as the most specific vector for transducing inner ear hair cells. Furthermore, we demonstrated that the loss of *Atp6v1b2* in hair cells leads to progressive cell death due to lysosomal dysfunction. Based on these findings, we explored a gene replacement strategy as a potential therapeutic approach.

## 2 Results

### 2.1 The *Atp6v1b2* Gene Plays an Essential Role in the Functionality of Cochlear HCs

**Figure 1:**
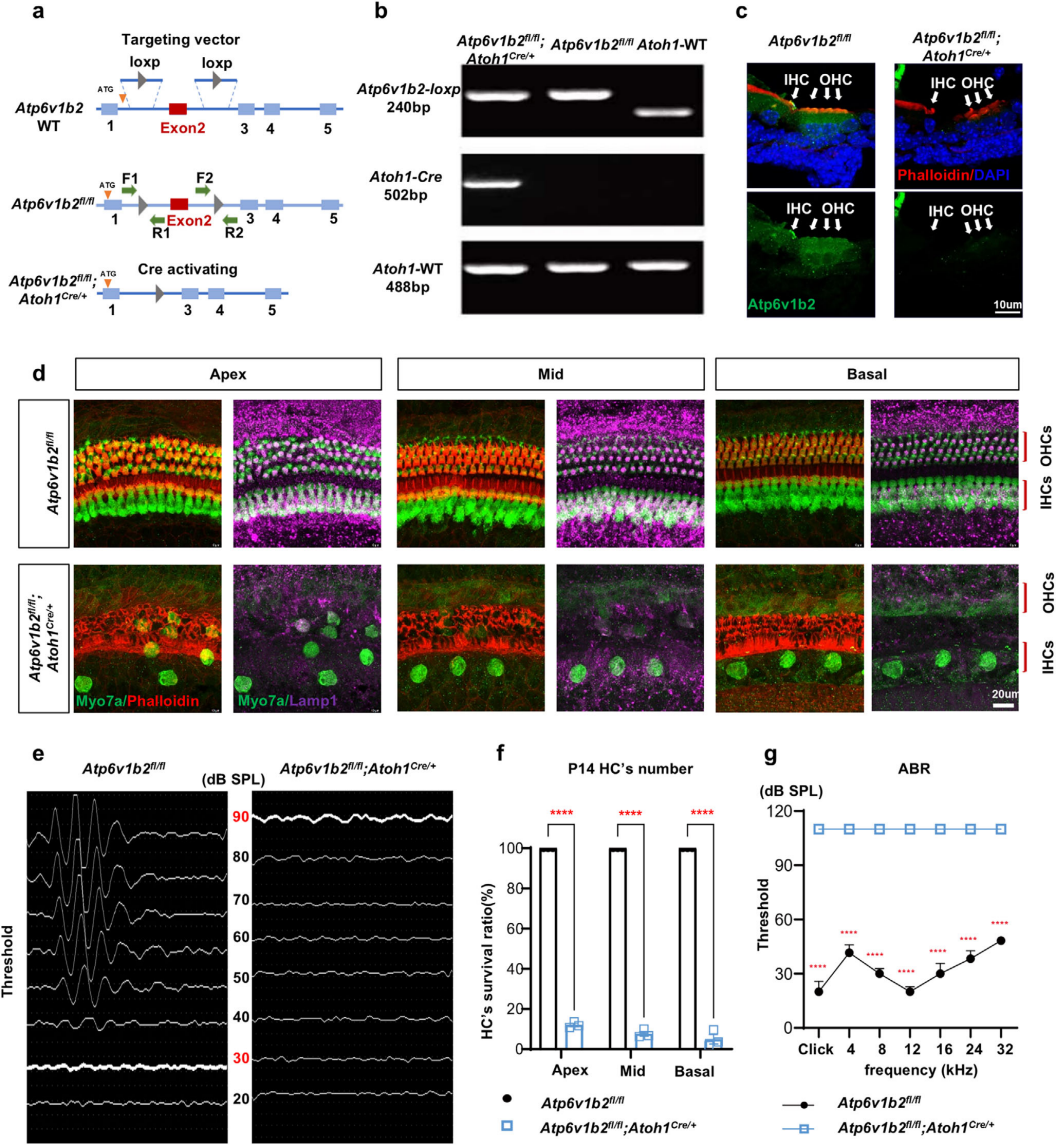
*Atp6v1b2* gene is indispensable for hair cell (HC) survival. a) Schematic diagram illustrated the generation of *Atp6v1b2* gene HC‐specific knockout mouse model, *Atp6v1b2^fl/fl^;Atoh1^Cre/+^*, by crossing *Atp6v1b2^fl/fl^* lines with *Atoh1^Cre/+^* lines. Successful integration of the loxp site was confirmed using two pairs of primers, F1‐R1 and F2‐R2, simultaneously. The translation initiation site ATG of Atp6v1b2 is located at the 58^th^‐60^th^ bases of Exon1. b) Gel electrophoresis of PCR products showed the genotyping of *Atp6v1b2^fl/fl^* and *Atp6v1b2^fl/fl^;Atoh1^Cre/+^* mice. On top, using the *Atp6v1b2*‐up primers, 197 base pairs (bp) of wild‐type (WT) sequence in WT mice and 240 bp of flox sequence in both *Atp6v1b2^fl/fl^* and *Atp6v1b2^fl/fl^;Atoh1^Cre/+^* mice were shown. In the middle, using primers specific for *Atoh1‐cre*, no corresponding band was detected in WT and *Atp6v1b2^fl/fl^* mice, whereas a 502 bp band was amplified in the *Atp6v1b2^fl/fl^;Atoh1^Cre/+^* mice carrying the *Atoh1‐ cre* sequence. At bottom, using primers for *Atoh1*, a 488 bp segment was amplified in WT, *Atp6v1b2^fl/fl^* and *Atp6v1b2^fl/fl^;Atoh1^Cre/+^* mice. c) The immunostaining of cochlea showed absence of Atp6v1b2 protein (green, indicated by white arrows) in both inner hair cells (IHCs) and outer hair cells (OHCs) in *Atp6v1b2^fl/fl^;Atoh1^Cre/+^* mice at P0. However, the presence of hair bundles (Phalloidin, red) indicated normal morphology of HCs. d) In comparison with *Atp6v1b2^fl/fl^* mice, the *Atp6v1b2^fl/fl^;Atoh1^Cre/+^* mice displayed reduced number of HCs (Myo7a, green), hair bundles (Phalloidin, red), and lysosomes (Lamp1, purple) at P14 (n = 10 ears per group). e) Click‐evoked auditory brainstem response (ABR) waveforms and thresholds revealed severe deafness in the *Atp6v1b2^fl/fl^;Atoh1^Cre/+^* mice (n = 12 ears per group). The hearing thresholds (dB SPL) were represented by bold white lines with corresponding numbers in red. f) At P14, *Atp6v1b2^fl/fl^;Atoh1^Cre/+^* mice demonstrated significant loss of HCs (n = 5 ears per group) in the apex, middle, and base of the cochlea. g) ABR thresholds for click stimuli at 4, 8, 12, 16, 24, and 32 kHz were compared between *Atp6v1b2^fl/fl^* and *Atp6v1b2^fl/fl^;Atoh1^Cre/+^* mice using two‐way ANOVA. *Atp6v1b2^fl/fl^;Atoh1^Cre/+^* mice showed lack of ABR thresholds at all frequencies compared to *Atp6v1b2^fl/fl^* mice. ^****^ *p* < 0.000l.

Although mutant mouse model derived from pathogenic variant of DDOD syndrome have been previously generated to mimic clinical patients,^[^ ^10^ ^]^ the role of the *Atp6v1b2* gene in HCs remained unclear due to functional compensation induced by premature termination codon (PTC) mutations in mice.^[^ ^17^ ^]^ To address this issue, we generated conditional knockout mice with a targeted deletion of the *Atp6v1b2* gene specifically in HCs. Intron regions were inserted at both ends of exon 2 of *Atp6v1b2*, flanked by *loxp* sequence, resulting in *Atp6v1b2*‐*loxp* (*Atp6v1b2^fl/fl^*) mice (**Figure** **1**a). These mice were then crossed with *Atoh1^Cre/+^* mice, which express Cre recombinase specifically in HCs, to generate *Atp6v1b2*‐HCs‐cKO (*Atp6v1b2^fl/fl^;Atoh1^Cre/+^*) mice. Successful gene deletion was confirmed at both the DNA and protein levels (Figure 1b,c). Notably, no ABR waveforms were detected in *Atp6v1b2^fl/fl^;Atoh1^Cre/+^* mice at P14, in contrast to *Atp6v1b2^fl/fl^* mice (Figure 1e,g). This severe to profound hearing loss phenotype is consistent with that observed in patients with *ATP6V1B2* variants. Morphological analysis of the cochlea at P14 revealed significant HC loss in *Atp6v1b2^fl/fl^;Atoh1^Cre/+^* mice compared to *Atp6v1b2^fl/fl^* mice (Figure 1d,f). Due to the nearly complete loss of outer hair cells (OHCs) by P14, DPOAE was not assessed. These findings underscore the critical role of *Atp6v1b2* in HC survival.

### 2.2 *Atp6v1b2^fl/fl^;Atoh1^Cre/+^* Mice Exhibit HC Morphological Abnormalities Due to Lysosomal Dysfunction

**Figure 2:**
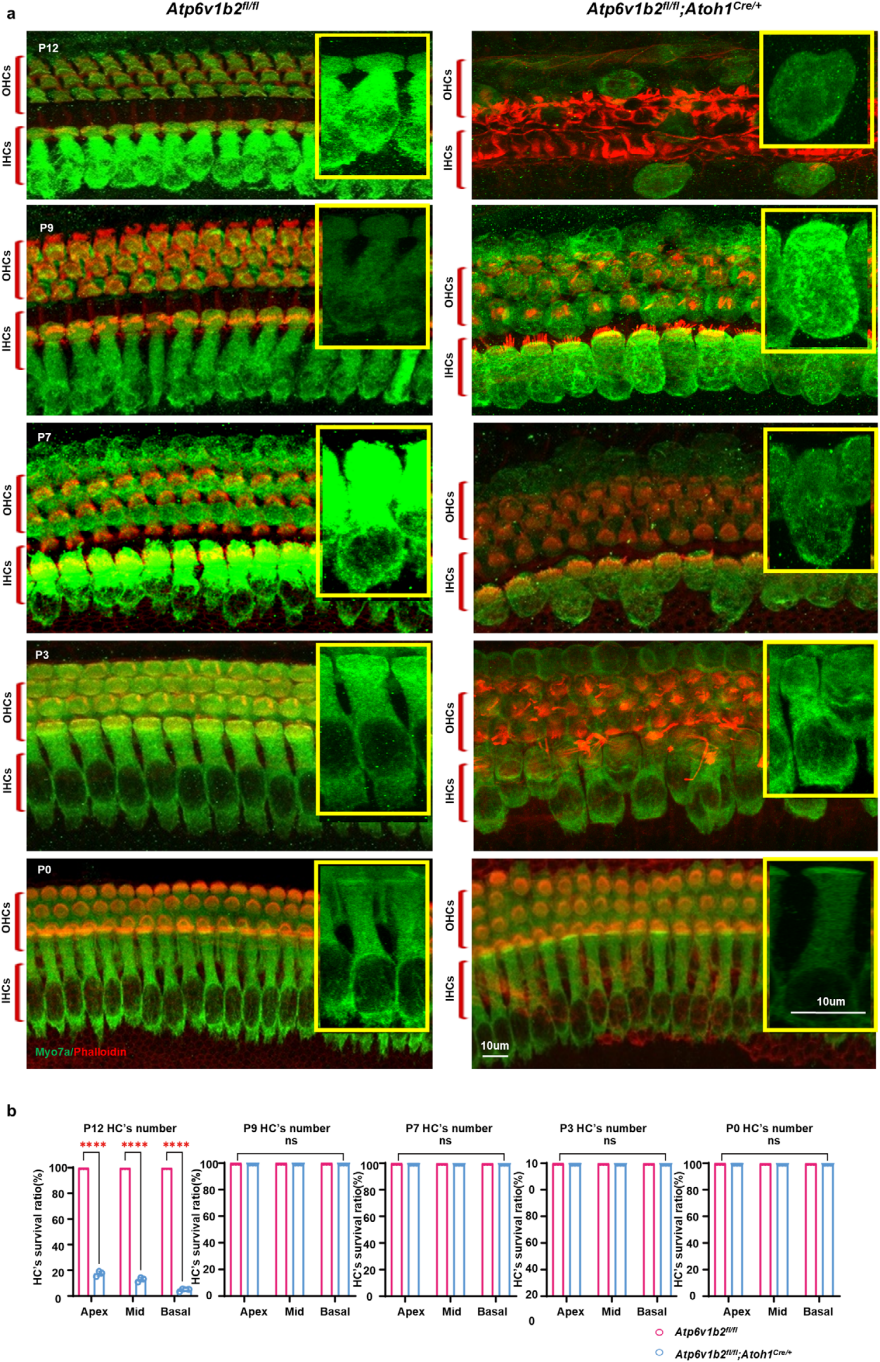
Pathological changes of hair cells (HCs) in *Atp6v1b2^fl/fl^;Atoh1^Cre/+^* mice at various postnatal time point. a) Representative confocal microscopy images showed the status of HCs in the cochlear middle gyrus of *Atp6v1b2^fl/fl^* control and *Atp6v1b2^fl/fl^;Atoh1^Cre/+^* mice. HCs and hair bundles were labeled with green (Myo7a) and red (Phalloidin) fluorescence, respectively. The diagram shown in the yellow frame on the right represents hair cell morphology under high magnification. In *Atp6v1b2^fl/fl^;Atoh1^Cre/+^* mice, no significant abnormalities were observed in the morphology and number of HCs’ cilia or bodies at P0, vacuole‐like changes within the HCs and abnormal morphology of cilia were observed at P3, visible swelling of the whole HCs appeared at P7, the morphological changes of the HCs further aggravated at P9, and loss of HCs became evident at P12. b) Quantification of HCs in *Atp6v1b2^fl/fl^* control (pink column) and *Atp6v1b2^fl/fl^;Atoh1^Cre/+^* (blue column) mice. Data are presented as means ±SEM (standard error of the mean). Statistical significance was evaluated using two‐way ANOVA (n = 10 ears per group). ^****^ *p* < 0.000l.

**Figure 3:**
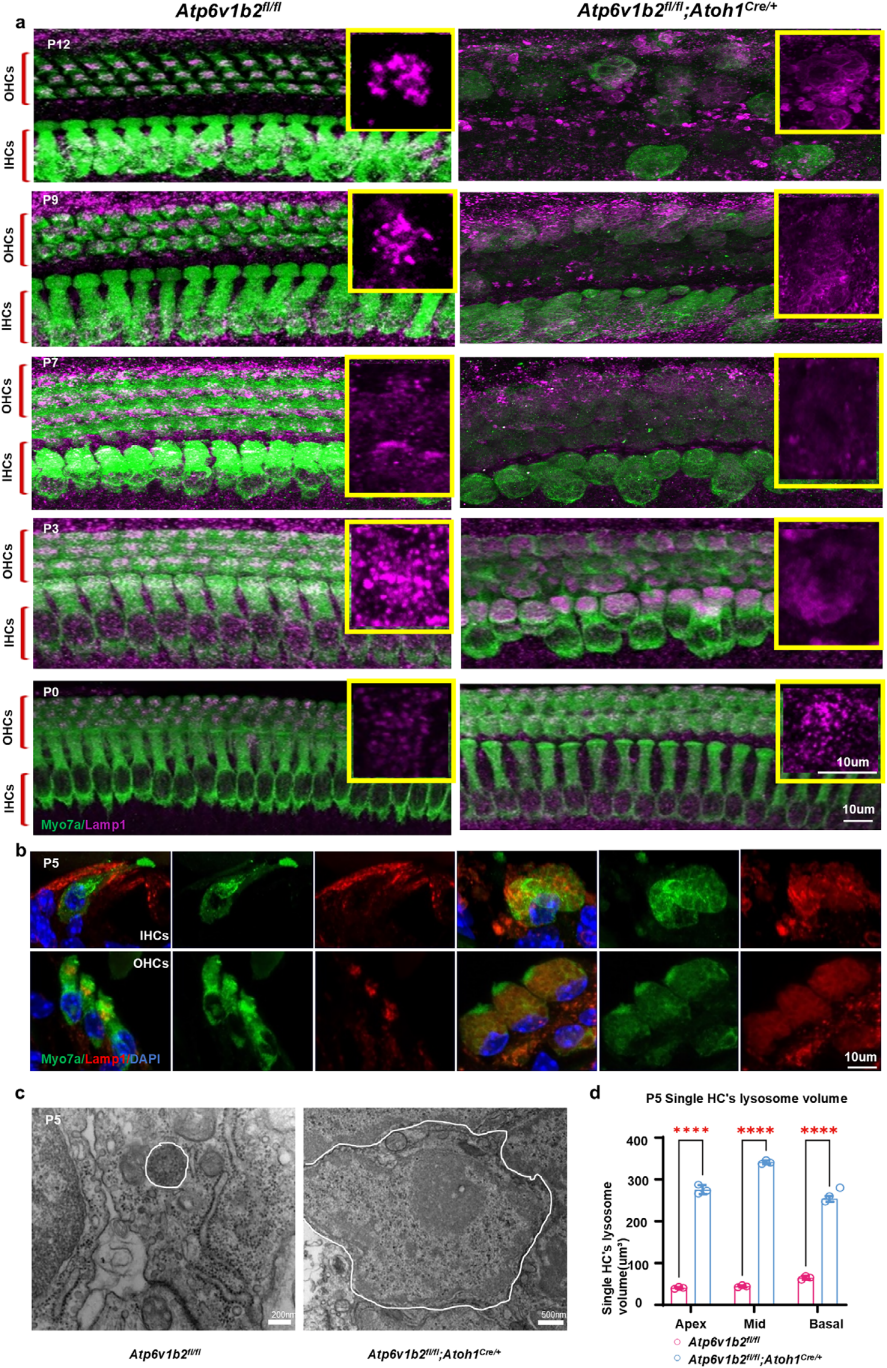
Lysosomal pathological alterations in hair cells (HCs) of *Atp6v1b2^fl/fl^;Atoh1^Cre/+^* mice. a) Representative images of HCs (Myo7a, green) and lysosomes (Lamp1, purple) in the cochlear middle gyrus of *Atp6v1b2^fl/fl^* control and *Atp6v1b2^fl/fl^;Atoh1^Cre/+^* mice were shown. The diagram shown in the yellow frame on the right represents a confocal image of lysosome inside a hair cell under high magnification. The lysosomes in HCs aggregated into clumps at P0, became swollen at P3, and persisted to P12 when the number of HCs decreased significantly in *Atp6v1b2^fl/fl^;Atoh1^Cre/+^* mice. b) Representative cross‐sectional images of lysosomes (Lamp1, red) within cochlear HCs (Myo7a, green) showed vacuolar changes in *Atp6v1b2^fl/fl^;Atoh1^Cre/+^* mice at P5. c) Representative transmission electron microscope (TEM) images revealed the lysosomes within HCs in *Atp6v1b2^fl/fl^;Atoh1^Cre/+^* mice were significantly enlarged compared to those in *Atp6v1b2^fl/fl^*. The white circles indicated lysosomes. d) The average lysosome volume was calculated and quantified in each HC (n = 80 HCs) of *Atp6v1b2^fl/fl^* control (pink column) and *Atp6v1b2^fl/fl^;Atoh1^Cre/+^* (blue column) mice. Statistical analysis revealed significant increase in lysosome volume happened at P5 in *Atp6v1b2^fl/fl^;Atoh1^Cre/+^* mice (n = 6 ears). Data are presented as means ±SEM. Statistical significance was evaluated using two‐way ANOVA. ^****^ *p* < 0.000l.

The deletion of *Atp6v1b2* is clearly associated with HC loss. To assess the potential role of gene therapy in preventing HC structural and functional loss, we examine HC morphology in *Atp6v1b2^fl/fl^;Atoh1^Cre/+^* mice from birth, as cellular survival is a prerequisite for successful gene therapy.^[^ ^18^ ^]^ Encouragingly, at P0, no significant abnormalities in HC morphology or number were observed (**Figure** **2**; Figure S1, Supporting Information). However, by P3, vacuole‐like changes appeared in HCs, followed by visible swelling at P7, further morphological deterioration at P9, and eventual HC loss by P12 (Figure 2; Figure S1, Supporting Information). We investigated the underlying cause of this progressive HC loss and found that lysosomes in HCs of *Atp6v1b2^fl/fl^;Atoh1^Cre/+^* mice aggregated into clumps at P0 (**Figure** **3**a; Figure S2, Supporting Information) and exhibited vacuolar changes with abnormal cilia morphology by P3 (Figure 3b), indicating a time‐dependent degenerative process. TEM revealed significantly enlarged cavities in HCs of *Atp6v1b2^fl/fl^;Atoh1^Cre/+^* mice at P5, due to the accumulation of degradation substrates (Figure 3c). Statistical analysis confirmed a significant increase in lysosomal volume in single HC from P0 onward (Figure 3d; Figure S3, Supporting Information). These findings suggest that significant HC loss does not occur within the first 9 days after birth (Figure 2b) and that lysosomal dysfunction play a crucial role in driving progressive HC loss in *Atp6v1b2^fl/fl^;Atoh1^Cre/+^* mice, surpassing the effect of the *Atp6v1b2* c.1516 C>T variant on lysosomal function.^[^ ^10b^ ^]^

*Atoh1* is expressed not only in cochlear HCs but also in vestibular hair cells (VHCs).^[^ ^19^ ^]^ At P14, VHCs in *Atp6v1b2^fl/fl^;Atoh1^Cre/+^* mice exhibited similar but less severe damage compared to cochlear HCs (Figure S4, Supporting Information). Lysosomes in VHCs also exhibited significant vacuole‐like changes (Figure S4, Supporting Information). This milder damage to VHCs aligns with the absence of overt vestibular symptoms in clinical patients.

### 2.3 Utilizing a Hair Cell‐Specific Promoter for the Delivery of Target Genes via AAV as the Vector Presents a Secure and Efficient Strategy

**Figure 4:**
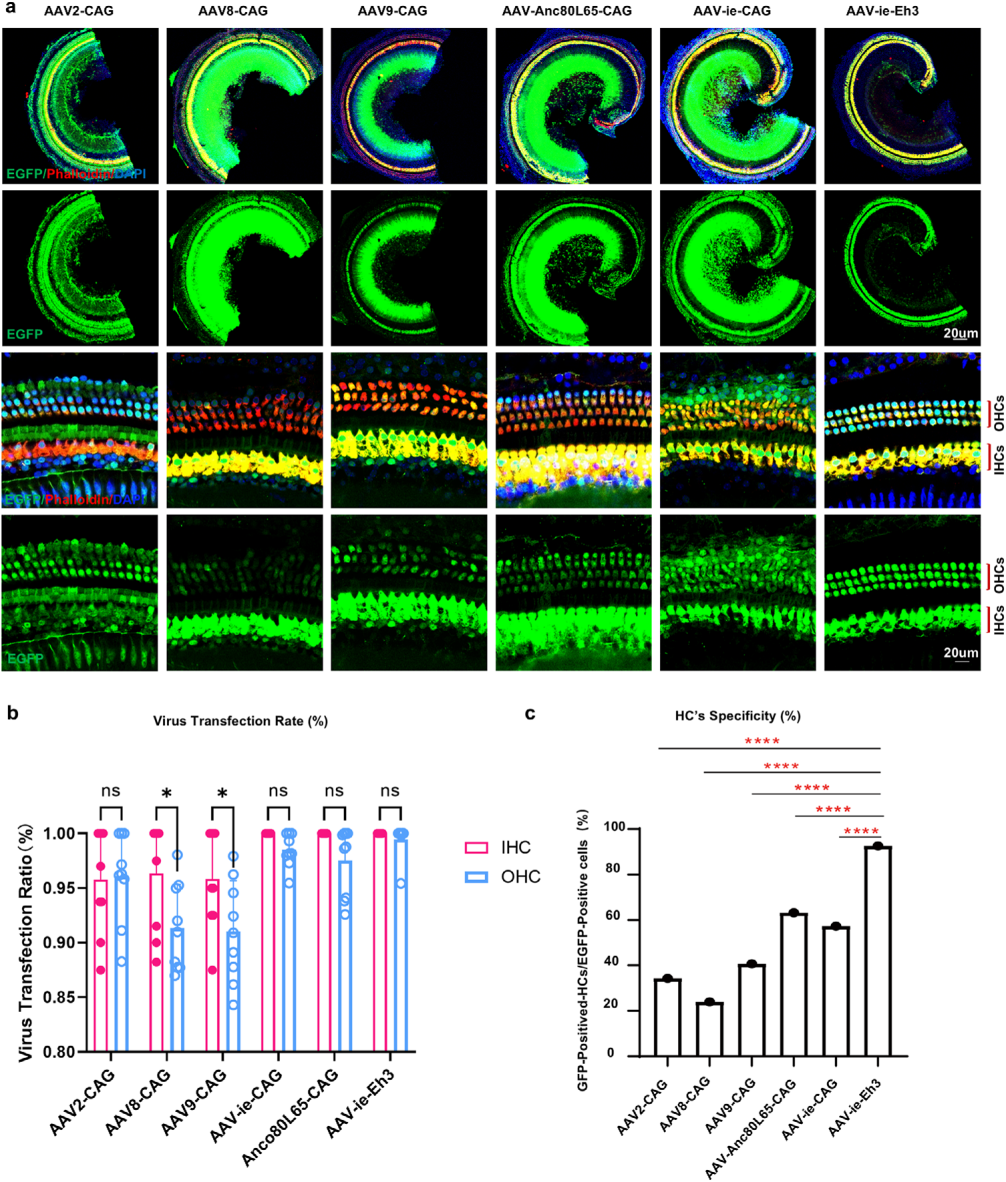
AAV‐ie with Enhancer3 promoter (AAV‐ie‐Eh3) is an optimal vector for gene therapy targeting hair cells (HCs). a) AAV2, AAV8, AAV9, AAV‐ie, and AAV‐Anco80L65 vectors expressing EGFP protein with CAG as the promoter, AAV‐ie vector with Enhancer3 as the promoter (AAV‐ie‐Eh3) were injected into the cochlea of WT mice at P0‐P2. At the 7th day post‐injection, the basilar membranes were dissected and antibody was used to label Myo7a (red). AAV‐ie‐Eh3 not only exhibited high efficiency toward HCs but also showed minimal EGFP expression in other cell types. b) Percentage analysis revealed no significant difference between EGFP‐ positive inner hair cells (IHCs, pink column) and outer hair cells (OHCs, blue column) among the different viral serotypes tested (n = 9 ears per group). c) The ratio of HCs to all EGFP‐ positive cells on the basilar membrane under high magnification was calculated to assess the specificity of viral infection (n = 9 ears per group). AAV‐ie‐Eh3 showed the highest efficiency in HCs infection and the lowest toxicity in the other cell types. Data are presented as means ±SEM. Statistical significance was evaluated using two‐way ANOVA. ^****^ *p* < 0.000l.

To explore gene therapy for *Atp6v1b2^fl/fl^;Atoh1^Cre/+^* mice, we evaluated AAV serotypes with high infectivity in inner ear HCs, including AVV2, AAV8, AAV9, AAV‐ie, and AAV‐Anc80L65.^[^ ^20^ ^]^ All serotypes carried the EGFP reporter under the control of the CAG promoter. Cochlea injections in P0‐P2 WT mice followed by observation of HC infections 1 week later.^[^ ^21^ ^]^ The findings revealed varying infection rates in HCs, with AAV‐ie and AAV‐Anc80L65 showing the highest infectivity (**Figure** **4**a,b). However, these viruses also exhibited off‐target expression in non‐HCs, leading to cytotoxicity. To address this issue, we replaced the CAG promoter with the HC‐specific Eh3 promoter, derived from the *Rbm24* gene,^[^ ^22^ ^]^ and integrated it with EGFP into AAV‐ie. This construct, AAV‐ie‐Eh3‐EGFP, demonstrated high HC‐specific infectivity with minimal off‐target expression (Figure 4a–c). Based on these results, we selected AAV‐ie‐Eh3 as the vector for targeted gene therapy in *Atp6v1b2^fl/fl^;Atoh1^Cre/+^* mice.

### 2.4 The Cochlear Scala Media Injection Demonstrated Significant Efficacy in Enhancing Both Auditory and Vestibular Functions in *Atp6v1b2^fl/fl^;Atoh1^Cre/+^* Mice

**Figure 5:**
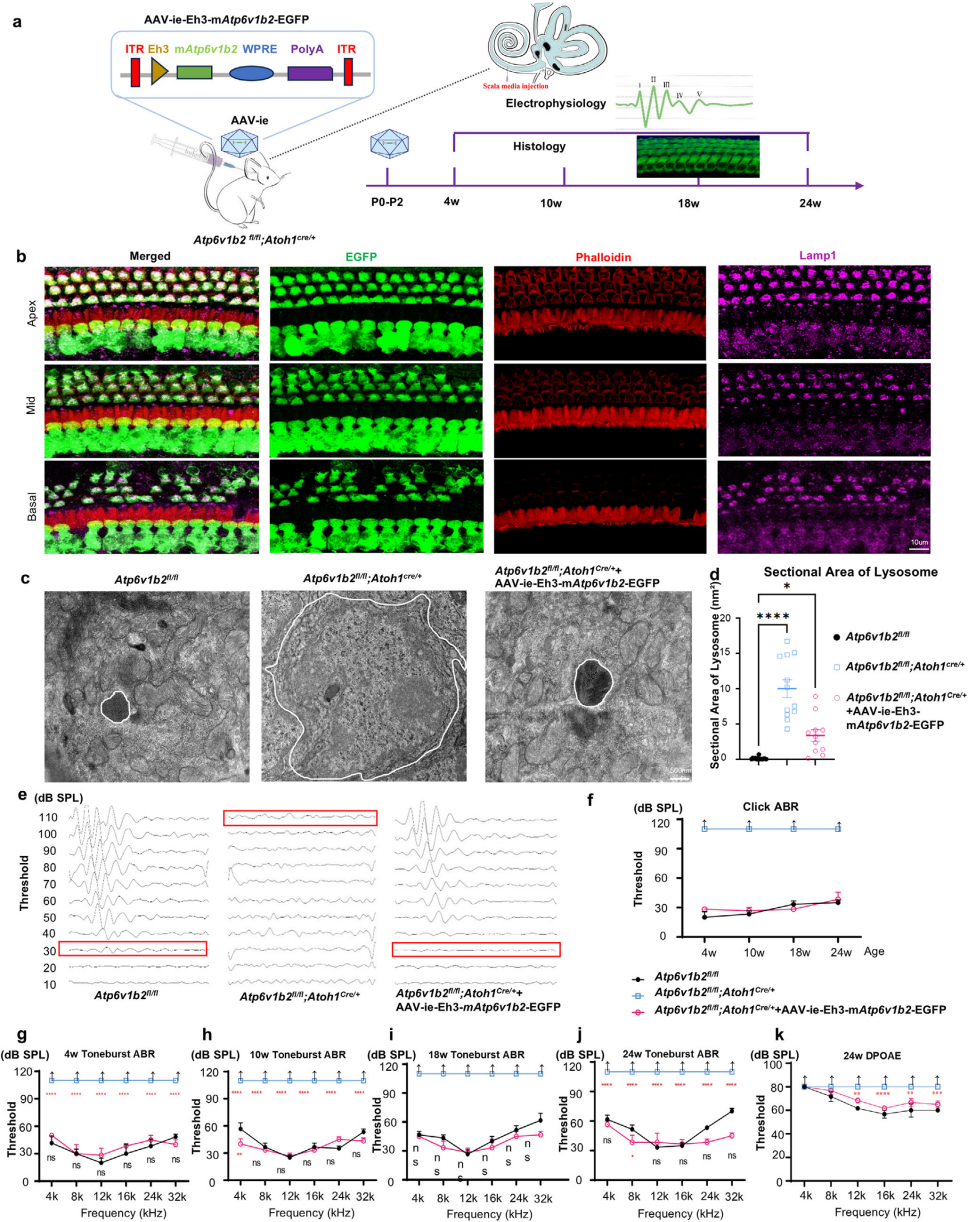
The auditory function and morphology of HCs in Atp6v1b2fl/fl;Atoh1Cre/+ mice were effectively restored for an extended duration through gene replacement. a) Schematic diagram for in vivo delivery of AAV‐ie‐Eh3‐m*Atp6v1b2*‐EGFP into the mouse cochlea. The administration of gene replacement was performed in neonatal *Atp6v1b2^fl/fl^;Atoh1^Cre/+^* mice aged between P0 and P2 through the approach of cochlear scala media injection. b) AAVie‐Eh3‐m*Atp6v1b2*‐EGFP injected HCs (EGFP, green), hair bundles (Phalloidin, red), and lysosomes (Lamp1, purple) were shown. Fourteen days after treatment, well‐preserved morphology of HCs’ bodies, cilia, and intracellular lysosomes were observed in virus‐infected HCs. c) The white circle on the TEM image highlighted the location of a wild type lysosome, mutant lysosome, and restored lysosome in the HCs, respectively. There was no excessive accumulation of degradation substrates in the lysosomes within HCs of the treated *Atp6v1b2^fl/fl^;Atoh1^Cre/+^* mice. d) The sectional area of lysosome was calculated and quantified in HCs’ lysosome of *Atp6v1b2^fl/fl^* control (black, n = 13), *Atp6v1b2^fl/fl^;Atoh1^Cre/+^* (blue, n = 12) and rescued *Atp6v1b2^fl/fl^;Atoh1^Cre/+^* (pink, n = 11) mice. Statistical analysis revealed significant increase in the sectional area of lysosomal in *Atp6v1b2^fl/fl^;Atoh1^Cre/+^* mice aged P7. Data are presented as means ±SEM. Statistical significance was evaluated using one‐way ANOVA. ^****^ *p* < 0.000l. e) Typical ABR waveforms for pre‐ and post‐gene therapy. f–j) The statistical analysis of ABR demonstrated the significant therapeutic effects involving click and toneburst last for at least 24 weeks after gene replacement (n = 6 ears per group). k) DPOAE remained almost the same as that in the control group 24 weeks after gene therapy in *Atp6v1b2^fl/fl^;Atoh1^Cre/+^* mice (n = 6 ears per group). Data are presented as means ±SEM. Statistical significance was evaluated using two‐way ANOVA. ^*^ *p* < 0.05, ^**^ *p* < 0.01, ^***^ *p* < 0.001, and ^****^ *p* < 0.000l.

After identifying AAV‐ie‐Eh3 as an effective vector, we performed gene therapy on *Atp6v1b2^fl/fl^;Atoh1^Cre/+^* mice. Key aspects of our approach included: First, targeting P0‐P2 mice to intervene before significant HC loss. Second, utilizing scala media injection for prolonged drug retention in the endolymph^[^ ^23^ ^]^ (**Figure** **5**a). Lastly, evaluating outcomes via ABR, DPOAE, and HC lysosomes morphology.

At 14 days post‐injection, HCs, cilia, and lysosomes were well‐preserved in treated mice (Figure 5b). TEM confirmed the absence of excessive lysosomal substrate accumulation (Figure 5c,d). There was no significant difference in hearing thresholds of ABR between treated *Atp6v1b2^fl/fl^;Atoh1^Cre/+^* mice, and *Atp6v1b2^fl/fl^* mice for Click and Tone‐burst stimuli (Figure 5e–k). Surprisingly, treated mice exhibited lower thresholds at 24 and 32 kHz compared to controls. The DPOAE results confirmed functional recovery of OHCs in treated mice (Figure 5k).

**Figure 6:**
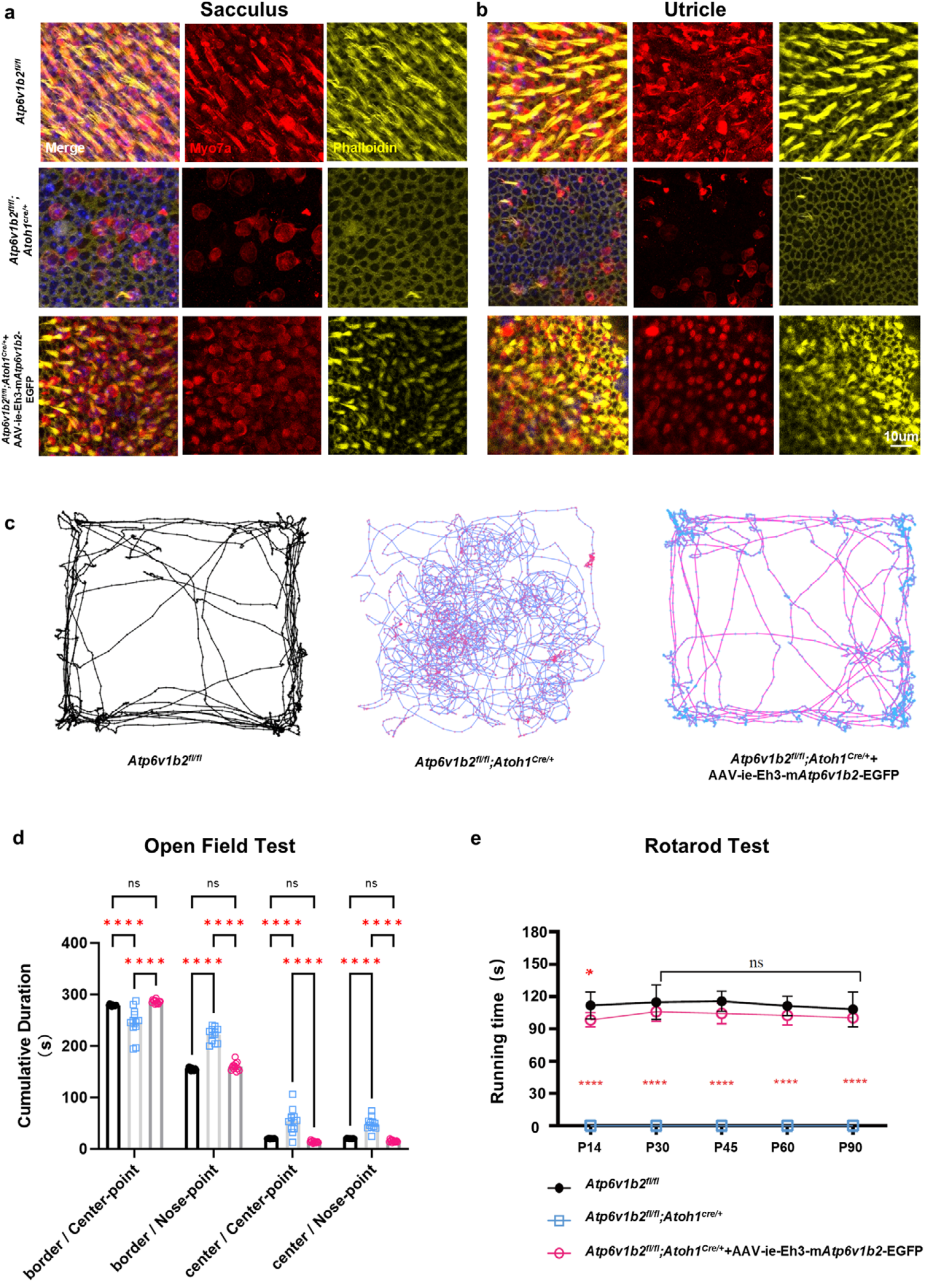
The vestibular function and cell morphology of Atp6v1b2fl/fl;Atoh1Cre/+ mice were effectively restored for an extended duration through gene replacement. a,b) In *Atp6v1b2^fl/fl^;Atoh1^Cre/+^* mice at P30, the immunostaining of VHCs exhibited significant loss. Thirty days after treatment, restoration of morphology of the VHCs’ bodies and cilia within the utricle and sacculus in *Atp6v1b2^fl/fl^;Atoh1^Cre/+^* mice were shown. c,d) The action trajectory map (c) and corresponding statistical analysis (d) of the mice in the open field test revealed no significant disparity in the movement patterns between *Atp6v1b2^fl/fl^;Atoh1^Cre/+^* and *Atp6v1b2^fl/fl^* control mice (n = 12 mice). e) The statistical results of the rotarod test revealed a significant enhancement in the balance function of *Atp6v1b2^fl/fl^;Atoh1^Cre/+^* mice after gene replacement (n = 12 mice). Data are presented as means ±SEM. Statistical significance was evaluated using two‐way ANOVA. ^*^ *p* < 0.05, ^**^ *p* < 0.01, ^***^ *p* < 0.001, and ^****^ *p* < 0.000l.

Given the scala media injection method, which facilitates drug accumulation in the endolymph where VHCs reside,^[^ ^24^ ^]^ we also assessed vestibular function in treated *Atp6v1b2^fl/fl^;Atoh1^Cre/+^* mice (**Figure** **6**). At 14 days post‐injection, VHC numbers significantly recovered, with restored lysosomal morphology and EGFP expression in transduced VHCs of the utricle and sacculus (Figure S5, Supporting Information). By 30 days post‐injection, VHCs and cilia in the sacculus and utricle of treated *Atp6v1b2^fl/fl^;Atoh1^Cre/+^* mice were well‐preserved (Figure 6a,b). To further investigate the recovery of balance function, we performed the open‐field and rotarod tests (Figure 6c–e). In the open‐field test, the frequency of circling behavior in the treated mice was significantly reduced, nearly eliminating all circling, and leaving only minor head bobbing behavior (Figure 6c,d). In the rotarod test, all treated mice spent more time running on the rotating rod compared to the untreated mice, which were unable to remain on the rotarod (Figure 6e). The treated mice locomoted similarly to the best‐performing *Atp6v1b2^fl/fl^* animals, indicating recovered vestibular function.

### 2.5 Safety Assessment of AAV‐ie‐Eh3‐*Atp6v1b2* Injection

AAV‐ie‐Eh3‐*Atp6v1b2* (1E+10^13^GC/ml, 1× dose per ear) was injected into P0‐P2 mice via the scala media. Histopathological evaluation at 4 weeks post‐injection revealed no morphological or quantitative changes in HCs (Figure S6a,b, Supporting Information). ABR thresholds were unaffected (Figure S6c, Supporting Information), and no significant differences in body weight, blood cell counts, or biochemical indicators were observed (Figure S6d and Tables S1 and S2, Supporting Information). Histological analysis of major organs showed no abnormalities (Figure S6e, Supporting Information). These results confirm the safety of AAV‐ie‐Eh3‐*Atp6v1b2* at therapeutic dose.

## 3 Discussion

ATP6V1B2 plays a critical role in hair cell function and is essential for hearing. In 2014, our group identified *ATP6V1B2* as the gene responsible for DDOD syndrome and provided evidence supporting haploinsufficiency as the underlying genetic mechanism.^[^ ^6^, ^10^, ^25^ ^]^

Genetically engineered animals are invaluable for studying the pathogenesis of human diseases and exploring potential treatments. Previously, we developed a knockin mouse model carrying the *Atp6v1b2* c.1516C>T (p.Arg506*) variant, the most common pathogenic variant identified in patients with DDOD syndrome. While these knockin mice exhibited progressive hearing loss, they did not fully replicate the congenital hearing loss observed in human patients.^[^ ^10a,b^ ^]^ Pathological changes were observed in the spiral ganglion, but no significant abnormalities were detected in hair cells. Further investigation revealed that *Atp6v1b1* compensates for the impaired function of *Atp6v1b2* in hair cells.^[^ ^10b^ ^]^ The fact that hearing loss in patients can be mitigated by cochlear implants underscores the importance of *ATP6V1B2* in hair cell function. To further investigate interventions for *Atp6v1b2*‐associated hearing loss, we established a hair cell‐specific conditional knockout mouse model (*Atp6v1b2* ^fl/f^ *;Atoh1^Cre/+^*) that accurately recapitulates the congenital severe to profound hearing loss phenotype seen in DDOD syndrome patients. To determine the optimal timing for intervention, we analyzed the morphology of hair cells and lysosomes from P0 to P12 using immunofluorescence staining and TEM in *Atp6v1b2^fl/fl^;Atoh1^Cre/+^* mice. No abnormalities were observed in inner or outer hair cells at P0. However, swollen and bubbling hair cells were evident by P3, and a significant reduction in hair cell numbers was noted by P12. Based on these findings, we selected the early neonatal period (P0‐P2) for intervention to preserve hair cell integrity.

The choice of gene therapy strategies for hearing loss depends on the nature of the causal variants and their functional consequences. For haploinsufficient variants, gene replacement is a viable approach. We delivered a functional, wild‐type version of the mouse *Atp6v1b2* cDNA into the inner ear of *Atp6v1b2^fl/fl^;Atoh1^Cre/+^* mice. AAVs are currently the preferred vectors for therapeutic gene delivery to the inner ear. After screening five AAV serotypes, we confirmed that AAV‐ie exhibited the highest transduction efficiency in the mouse inner ear, consistent with previous studies.^[^ ^16^ ^]^ In 2022, Tao et al. reported a novel AAV‐ie mutant, AAV‐ie‐K558R, which efficiently transduces hair cells and supporting cells.^[^ ^26^ ^]^ However, in mice, AAV‐ie mediated reporter gene expression was observed in various cochlear cell types, including hair cells, supporting cells, and the stria vascularis, indicating limited specificity. To address this, we developed strategies to modify the AAV‐ie promoter to target expression specifically to HCs. We demonstrated that AAV‐ie incorporating the *Rbm24* enhancer 3 and *Hsp68* promoter (AAV‐ie‐Eh3) drives robust and specific expression of therapeutic cDNA in transduced hair cells. AAV‐ie‐Eh3 proved to be a highly effective vector for targeted expression in inner and outer hair cells, offering potential for treating hair cell‐specific hearing loss. Delivery of wild‐type *Atp6v1b2* cDNA via AAV‐ie‐Eh3 through scala media injection rescued severe hearing loss, restoring auditory function to normal levels. The treated mice exhibited preserved hair cell quantity and function, as well as normal lysosomal morphology and function, compared to untreated mice.

The durability of hearing improvement is a critical factor for translating preclinical findings into clinical applications. However, many proof‐of‐concept studies reported a decline or loss of the therapeutic effects within 2 months of intervention.^[^ ^18^ ^]^ In contrast, our results demonstrated that a single administration of AAV gene replacement therapy in neonatal mice restored ABR thresholds to normal levels across all tested frequencies. This therapeutic effect remained stable for at least 6 months post‐intervention. The long‐term efficacy can be attributed to the use of a hair cell‐specific promoter, which minimizes off‐target effects and ensures sustained expression of the therapeutic gene in hair cells.

Notably, the human cochlea is well‐advanced by mid‐gestation and fully matures at birth, whereas in mice, cochlear development begins at E11.5 and is not complete until P14.^[^ ^27^ ^]^ In this study, neonatal P0‐P2 *Atp6v1b2*‐HCs‐cKO (*Atp6v1b2^fl/fl^;Atoh1^Cre/+^*) mice with immature cochlea morphology were injected with AAV‐ie‐Eh3‐m*Atp6v1b2*, successfully preventing hair cell loss and restoring lysosome morphology. It is important to note that DDOD syndrome patients exhibit variable degrees of hearing loss, ranging from moderate to severe. The presence of functional hair cells in the inner ear after birth is crucial for the potential rescue or restoration of hearing. Consequently, gene replacement therapy may offer a promising therapeutic opportunity for DDOD syndrome patients.

A limitation of this study is the need for further consideration before translating these proof‐of‐concept findings into human treatments, given the development differences between mouse and human auditory systems. Nonetheless, the encouraging results from our *Atp6v1b2*‐HCs‐cKO (*Atp6v1b2^fl/fl^;Atoh1^Cre/+^*) mouse models provide a strong foundation for future research.

## 4 Conclusion

We established and validated a hair cell‐specific conditional knockout mouse model for *Atp6v1b2*, which exhibited progressive and severe cochlear degeneration beginning shortly after birth. This degeneration narrows the window for effective intervention to the first few postnatal days. Using the AAV‐ie‐Eh3 delivery system, we achieved robust and specific expression of *Atp6v1b2* in hair cells. Restoration of *Atp6v1b2* expression effectively ameliorated hair cell morphological abnormalities and repaired lysosomal dysfunction. The therapeutic effects persisted for at least 6 months post‐intervention. Furthermore, we demonstrated that AAV‐ie‐Eh3 is a highly effective vector for targeting hair cells in mice, highlighting its potential as a therapeutic tool for treating hereditary hearing loss caused by hair cell‐specific genetic variants. This study provides significant insights into the pathophysiological mechanisms underlying *ATP6V1B2*‐associated hearing loss in vivo. These findings pave the way for the development of gene replacement therapies for the inner ear, offering a promising approach to address hereditary hearing loss caused by specific genetic defects in hair cells.

## 5 Experimental Section

### Generation of Mouse Models

All experiments were conducted in accordance with protocols approved by the Ethics Committee of the Chinese PLA General Hospital. Conditional *Atp6v1b2* knockout mice (*Atp6v1b2^fl/fl^;Atoh1^Cre/+^*, cKO) and their littermates (*Atp6v1b2^fl/fl^*), which served as wild‐type (WT) controls, were utilized. The *Atp6v1b2^fl/fl^* mice were generated by the National Institute of Biological Sciences, Beijing (NIBS) on a C57BL/6J genetic background. PCR genotyping of the floxed *Atp6v1b2* allele was performed using the following primers: *Atp6v1b2*‐up‐F (5′‐GCCTTCTCTTTCGCAGTGTCTTTCC‐3′) and *Atp6v1b2*‐up‐R (5′‐CAAGGCTGCTTCCCTCTAAAAGTTCAG‐3′), *Atp6v1b2*‐down‐F (5′‐GAGAGATGTGGCATCTTATGTCCTTTGTG‐3′) and *Atp6v1b2*‐down‐R (5′‐GGGCTGTAAGGCAGTATTGAGACAATCC‐3′). The *Atoh1^Cre/+^* mice, also on a C57BL/6J background, were obtained from Shandong University. Genotyping for *Atoh1^Cre/+^* was performed using the primers: *Atoh1‐Cre* ‐F (5′‐GCGCAGCGCCTTCAGCAAC‐3′) and *Atoh1‐Cre* ‐R (5′‐GCCCAAATGTTGCTGGATAGT‐3′), and for *Atoh1‐*WT, using the following primers: *Atoh1‐*WT‐F (5′‐TGACGCCACAGCCACCTGCTA‐3′) and *Atoh1‐*WT‐R (5′‐GGACAGCTTCTTGTCGTTGTTG‐3′).

### Immunostaining

Cochlea were dissected from mice and fixed in 4% paraformaldehyde (PFA) for 2 h at room temperature. Decalcification was performed in 10% ethylenediaminetetraacetic acid (EDTA, pH = 7.2) at room temperature for ≈4 h (timing adjusted based on the age of the mice). The basilar membrane, saccular macula, and maculae utriculi were dissected in phosphate buffer (PBS). Primary antibodies included: Rabbit anti‐Myosin‐VIIa (Proteus Biosciences, Ramona, CA, United States, 1:400), rat anti‐Lamp1 (ab25245, Abcam, United Kingdom, 1:500), rabbit anti‐Atp6v1b2 (ab73404,Abcam, United Kingdom,1:400). Secondary antibodies included: goat anti‐rabbit IgG (A‐11008, Thermo Fisher Scientific, Waltham, MA, United States,1:400), goat anti‐rat IgG (A‐21247, Thermo Fisher Scientific, Waltham, MA, United States,1:400), Alexa Fluor 555 (A34055, Thermo Fisher Scientific, Waltham, MA, United States,1:600). Nuclei were counterstained with 4′,6‐diamidino‐2‐phenylindole (DAPI). All antibodies were diluted in PBS containing 0.2% Triton and 10% goat serum.

### Transmission Electron Microscopy (TEM)

Cochlea samples were perfused with 0.25% glutaraldehyde (in 0.1 m phosphate buffer) overnight at 4 °C, followed by decalcification in 10% EDTA (pH 7.2) at 4 °C for 2–4 days. After softening, the cochlea were dehydrated in ethanol and embedded in Araldite resin. Semi‐thin sections (70 nm) were prepared and stained for localization. Ultra‐thin sections were stained with uranyl acetate and lead citrate. TEM imaging was performed using a H‐7650B microscope. Non‐overlapping areas of the aramid nanofibers (ANF) cross‐sections were imaged at a magnification of 3400×. At least three mice per group were included in all analyses.

### Confocal Microscope

Confocal Z‐axis scanning (0.35 µm step size, 40× oil immersion) was performed on cochlear tissue using a Leica SP8 microscope. Images were processed using Leica SP8 software. At least three mice per group were included in immunofluorescence analyses.

### In Vivo Auditory Electrophysiological Evaluations

Auditory brainstem response (ABR) analysis were conducted on anesthetized mice (intraperitoneal injection of Avertin, 0.2ml/10g). The body temperature was maintained using an electric blanket. Recording electrodes were placed subcutaneously between the ears, with reference and ground electrode positioned at the auricle and the groin, respectively. ABR data were collected using BiosigRZ software controlled by an RZ6 workstation. Stimuli included click sounds (0.1 ms duration, 21 Hz) and pure‐tone bursts (4k‐32 kHz, 5 ms duration, Cos2 gating, 21 Hz). Sound levels ranged from 110 dB SPL to 10 dB in 10 dB steps. Responses were recorded for 10 ms at a 25 kHz sampling rate (filtered 100 Hz to 3 kHz), with 512 responses averaged per stimulus.

### Distortion‐Product Otoacoustic Emission (DPOAE)

DPOAE measurements were conducted under the same condition as ABR testing. Ipsilateral acoustic stimulation and simultaneous DPOAE measurement were performed using a TDT BioSigRZ system. Stimuli were synthesized at 200 kHz using SigGen software, with f2/f1 = 1.25, L1 = 65 dB, and L2 = 50 dB SPL. Sound level ranged from 30 to 80 dB SPL in 10 dB steps at frequencies of 4–32kHz. Responses were amplified 10000‐fold, band‐pass filtered (3–33 kHz), digitized, and averaged (256 responses).

### Evaluation of Vestibular Balance

Vestibular function was assessed using the open‐field test (OF) and rotarod test. The open‐field apparatus consisted of a 40cm×40cm×40 cm chamber. Mice aged 4–12 weeks were allowed to explore the chamber for 10 min, with behavior recorded using an overhead camera. Mice were acclimated to new cages daily for 5 days prior to testing.

For the rotarod test, mice were trained three times daily for 5 days. During testing, mice were placed on a rotarod at a constant speed of 5 RPM for 5 min. Each mouse underwent five trails, with a 3 h interval between trails. The time to fall was recorded, and the highest and lowest values were excluded before averaging. Data from mice exhibiting unstable behavior on intentional early termination were excluded.

### AAV Virus Production and Screening

All AAVs were custom‐produced by Packgene Biotech, Guangzhou, China. AAV2‐CAG, AAV8‐CAG, AAV9‐CAG, AAV‐ie‐CAG, and AAV‐Anc80L65‐CAG vectors were used to mediate enhanced green fluorescent protein (EGFP) expression. Viral titers were approximately 1E+13 genome copies per ml (GC/ml). The *Rbm24* enhancer 3 (Eh3), combined with the mouse heat shock protein 68 (Hsp68) mini‐promoter, was used to drive specific transgene expression in cochlear hair cells. A previous invention^[^ ^22^ ^]^ led to the synthesis of the enhancer 3 (Eh3) promoter, which was then compared with the CAG promoter in terms of its specificity and ability to drive transgene expression. This resulted in the development of a therapeutic agent for DDOD syndrome. The Eh3 promoter was used to replace the CAG promoter to construct the AAV‐ie‐Eh3‐m*Atp6v1b2*‐EGFP plasmid. Since virally‐mediated gene expression driven by the Eh3 promoter was identified to be more stable and specific in the cochlea compared to that driven by the CAG promoter,^[^ ^22^ ^]^ we constructed AAV‐ie‐Eh3‐m*Atp6v1b2‐*EGFP, with a titer of 1E+13 GC/ml. Virus titers were measured using qPCR. Virus aliquots (2 µl) were stored at −80 °C. EGFP expression was analyzed 6–7 days post‐injection.

### Mouse Cochlear Scala Media Injections

Postnatal days 0–2 (P0‐P2) pups were anesthetized using hypothermia on ice for 1.5–2.5 min. A post‐auricular incision exposed the otic bulla, and the cochlea was visualized under an asana microscope (RWD Taiwan 77008S). The injection site was identified at the intersection of the stapedial artery and facial nerve. A glass micropipette was used to deliver 500 nanoliters (nL) of virus at a rate of 400 nL min^−1^, controlled by a microsyringe pump (Old Sun Studio, China, LSS SMO‐10CP). Procedures were completed within 10 min, after which the incision was sutured. Pups were placed on a 37 °C heating pad for recovery and returned to their mother after they fully recovered. Standard post‐operative care was provided.

### Statistical Analysis

All experiments were conducted in triplicate using independent samples. Statistical analyses were performed directly on the raw data, as no pre‐processing steps (e.g., transformation, normalization, or outlier evaluation) were required. Differences among groups were determined using two‐way ANOVA followed by multiple comparisons, as indicated in the figure captions. The data were presented as the mean ± standard error of the mean (SEM). Statistical significance was set at *p* < 0.05, with the following designations: ^∗^ *p* < 0.05, ^∗∗^ *p* < 0.01, ^∗∗∗^ *p* < 0.001, ^∗∗∗∗^ *p* < 0.0001. GraphPad Prism 9.0 (GraphPad Software, San Diego, CA) was used for all statistical analyses.

### Image Processing Using Imaris

Quantitative analysis was performed using Imaris ×64 v9.8.0 software (Bitplane AG, Belfast, UK). For each immunofluorescent channel, three key measurements were quantified: i) counts of hair cells, lysosomes, or puncta; ii) per count and total (sum) volumes (µm^3^); and iii) total intensity of immunofluorescence (in arbitrary units). All measurements were adjusted for the field volume (µm^3^) of the corresponding region of interest within the acquired z‐stack.

### Ethics Approval and Consent to Participate

This study was approved by the Chinese People's Liberation Army General Hospital Research Ethics Committee (reference number S2016–120–02). All in vivo experiments were carried out in accordance with CALAS (Chinese Association for Laboratory Animal Science) guidelines for the care and use of laboratory animals and were approved by the Animal Care and Use Committees of Chinese PLA General Hospital.

## Conflict of Interest

W.X. is a co‐founder of SimpGen Therapeutics. The authors declare no conflict of interest.

## Author Contributions

G.W., S.Q., and X.G. contributed equally to this work. W.X., P.D., and Y.Y. conceived of the study and were responsible for submission of the manuscript for publication. G.W., S.Q., X.G., and Y.Y. participated in its design and drafting. G.W. and S.Q. performed the experiments. G.W., S.Q., L.Z., Y.C., Y.M., H.F., J.Y., G.D., H.N., and W.Z. participated in the literature search, data collection, and data analysis. All authors read and approved the final manuscript.

## Supporting information



Supporting Information

## Data Availability

The data that support the findings of this study are available in the supplementary material of this article.
