# Supplementary material for: Single Administration of AAV‐mAtp6v1b2 Gene Therapy Rescues Hearing and Vestibular Disorders Caused by Atp6v1b2‐Induced Lysosomal Dysfunction in Hair Cells
Source: Adv Sci (Weinh). 2025 Mar 11;12(29):2408878. doi: 10.1002/advs.202408878 (PMC12362768; doi:10.1002/advs.202408878)
Supplement: Supplementary file 1 — Supporting Information [file ADVS-12-2408878-s001.doc]

**
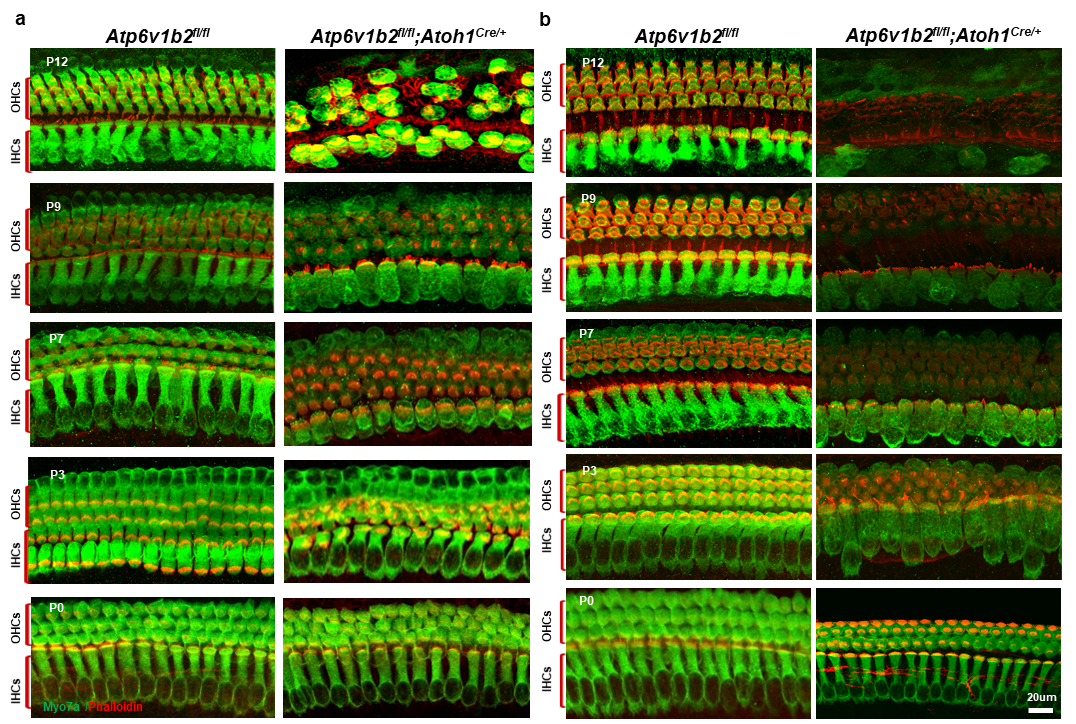
**

**Fig.S1 Pathological changes of hair cells (HCs) in *Atp6v1b2 ^fl/fl^;Atoh1^Cre/+^* mice at various postnatal time point.**

**a.** Representative confocal microscopy images showed the status of HCs in the cochlear apical gyrus of *Atp6v1b2^fl/fl^* control and *Atp6v1b2^fl/fl^*;*Atoh1^Cre/+^* mice. HCs and hair bundles were labeled with green (Myo7a) and red (Phalloidin) fluorescence, respectively. **b**. Representative confocal microscopy images showed the status of HCs in the cochlear basal gyrus of *Atp6v1b2^fl/fl^* control and *Atp6v1b2^fl/fl^*;*Atoh1^Cre/+^* mice. HCs and hair bundles were labeled with green (Myo7a) and red (Phalloidin) fluorescence, respectively. In *Atp6v1b2^fl/fl^*;*Atoh1^Cre/+^* mice, no significant abnormalities were observed in the morphology and number of HCs’ cilia or bodies at P0, vacuole-like changes within the HCs and abnormal morphology of cilia appeared at P3, and loss of HCs became evident at P12.

**
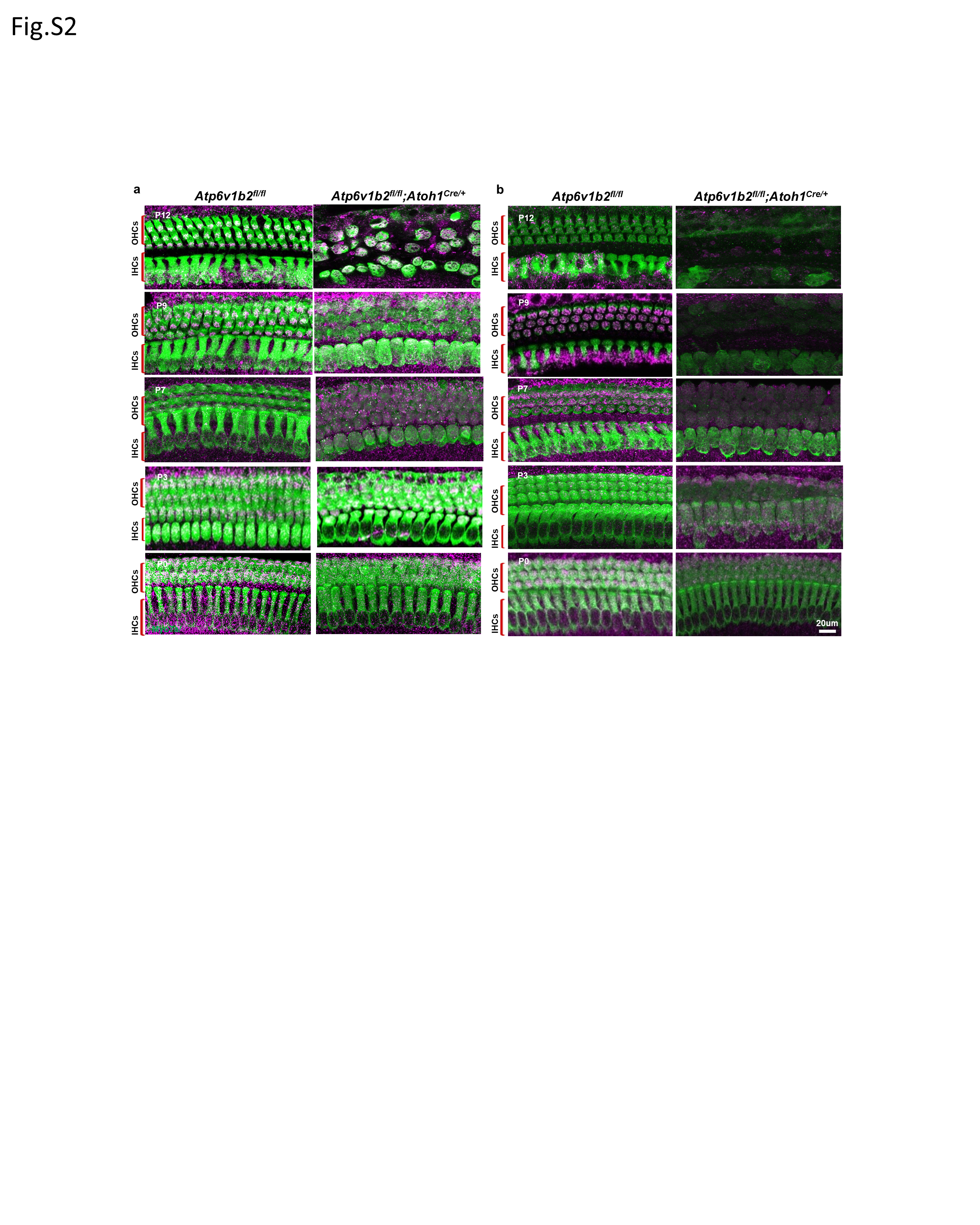
**

**Fig.S2 Lysosomal pathological alterations in hair cells (HCs) of *Atp6v1b2^fl/fl^;Atoh1^Cre/+^* mice.**

**a**.Representative images of HCs (Myo7a, green) and lysosomes (Lamp1, purple) in the cochlear apical gyrus of *Atp6v1b2^fl/fl^* control and *Atp6v1b2^fl/fl^*;*Atoh1^Cre/+^* mice were shown. **b**. Representative images of HCs (Myo7a, green) and lysosomes (Lamp1, purple) in the cochlear basal gyrus of *Atp6v1b2^fl/fl^* control and *Atp6v1b2^fl/fl^*;*Atoh1^Cre/+^* mice were shown. The lysosomes in HCs aggregated into clumps at P0, became swollen at P3 and persisted to P12 when the number of HCs began to decrease in *Atp6v1b2^fl/fl^*;*Atoh1^Cre/+^* mice.

**
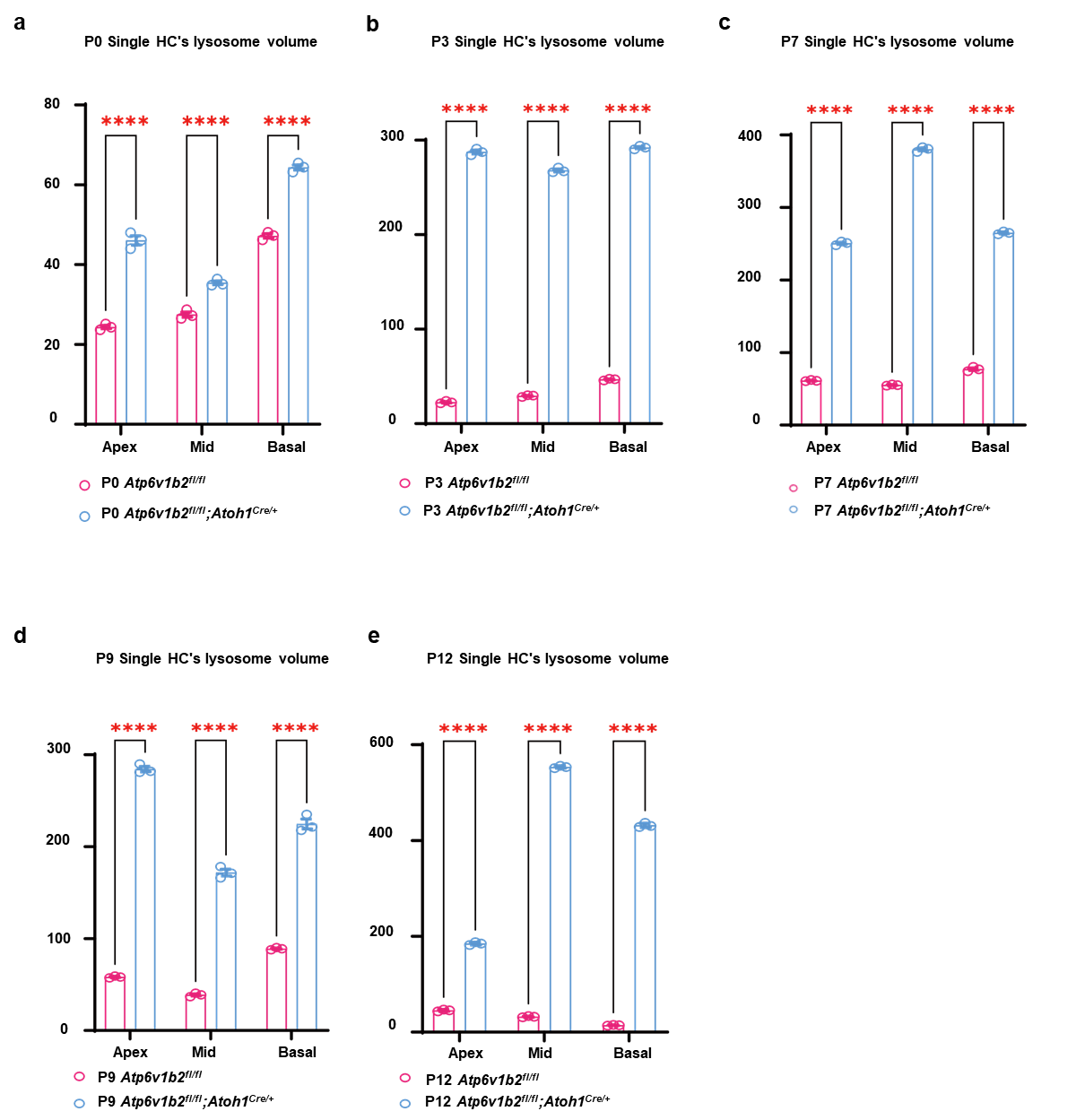
Fig.S3 Volume of lysosomes in a single hair cell (HC) of *Atp6v1b2 ^fl/fl^;Atoh1^Cre/+^* mice.**

**a-e**. The mean lysosome volume within one HC (n=80 hair cells) was calculated and quantified for *Atp6v1b2^fl/fl^* control (pink columns) and *Atp6v1b2^fl/fl^;Atoh1^Cre/+^* group (blue columns) mice at P0, P3, P7, P9 and P12, respectively. A statistical analysis using two-way ANOVA revealed a significant increase in lysosome volume in *Atp6v1b2^fl/fl^;Atoh1^Cre/+^* mice as early as postnatal day 0 (P0, n=6 ears). The p-value was less than 0.0001.

**
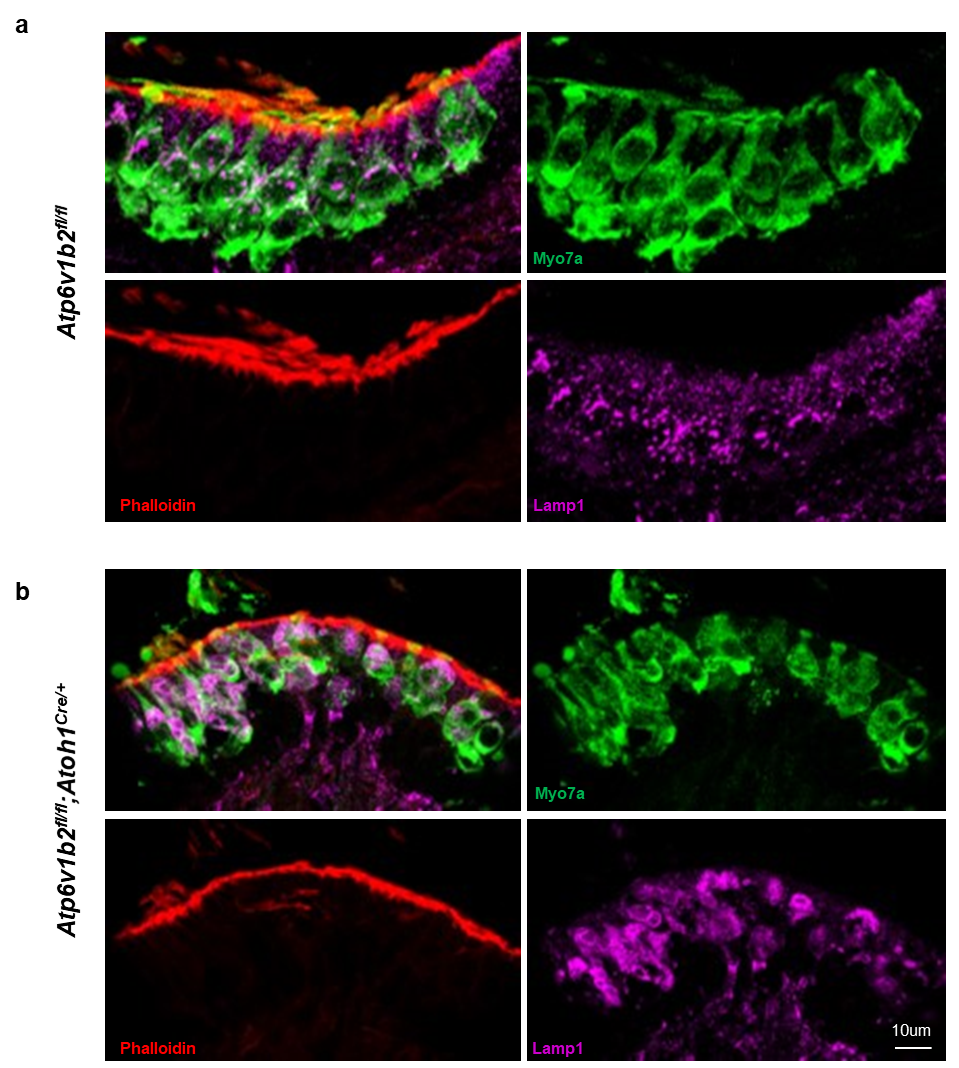
**

**Fig.S4** **Pathological changes in cochlear sections of vestibular hair cells (VHCs) from *Atp6v1b2 ^fl/fl^;Atoh1^Cre/+^* mice.**

**a-b**. Representative images of VHCs (Myo7a, green), lysosomes (Lamp1, purple) and hair bundle（phalloidin，red）in the cochlear cross-section of *Atp6v1b2 ^fl/fl^* control and *Atp6v1b2 ^fl/fl^;Atoh1^Cre/+^* mice were shown. In *Atp6v1b2 ^fl/fl^;Atoh1^Cre/+^* mice at P14, the immunostaining of lysosomes within VHCs exhibited significant vacuole-like changes but without obvious cell loss.

**
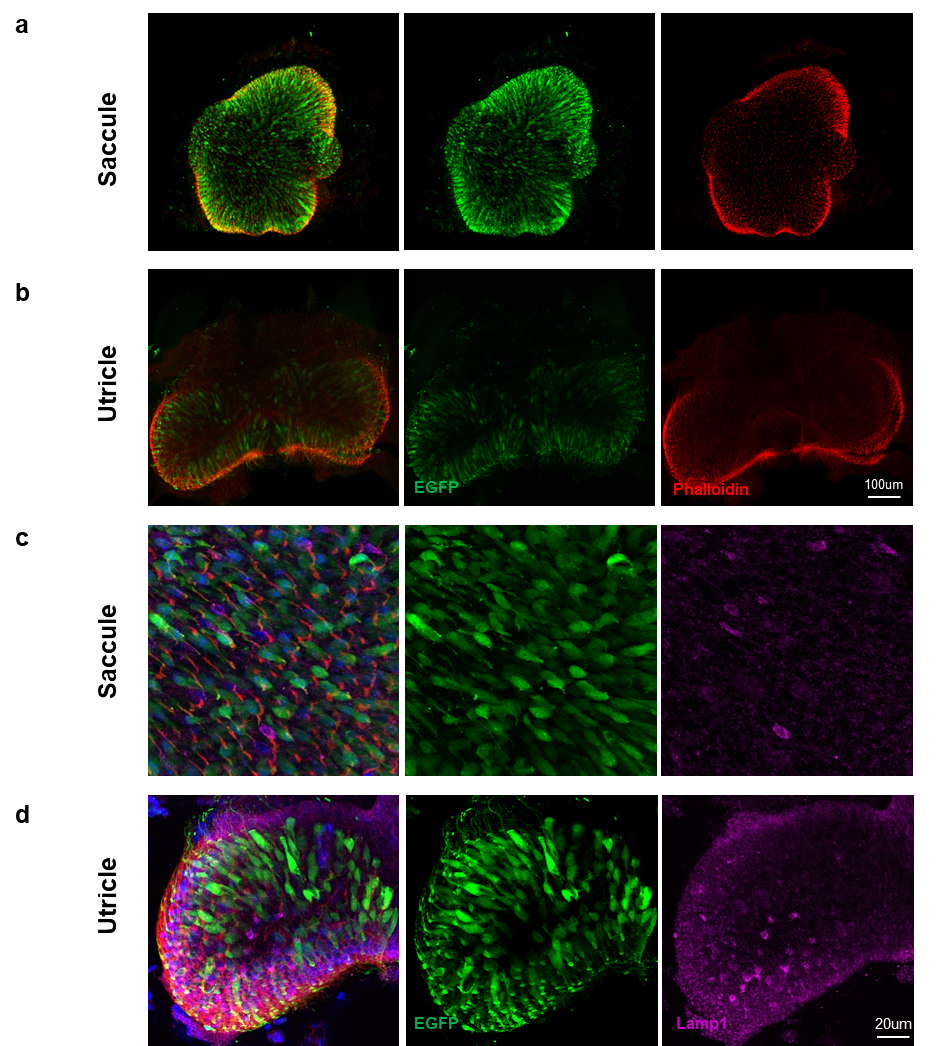
Fig.S5 AAV-ie‐Eh3-m*Atp6v1b2*-EGFP infected VHCs effectively and restored the morphology of lysosomes in *Atp6v1b2 ^fl/fl^;Atoh1^Cre/+^* mice.**

**a-b**. AAV-ie‐Eh3-m*Atp6v1b2*-EGFP injected VHCs (EGFP, green) and hair bundle (Phalloidin, red) were shown in the saccule and utricle. Fourteen days after treatment, the morphology of VHCs’ bodies and cilia was preserved. **c-d**. AAV-ie‐Eh3-m*Atp6v1b2*-EGFP injected VHCs (EGFP, green) and lysosomes (Lamp1, purple) were shown in the saccule and utricle. Fourteen days after treatment, well-preserved morphology of VHCs and intracellular lysosomes was observed.

**
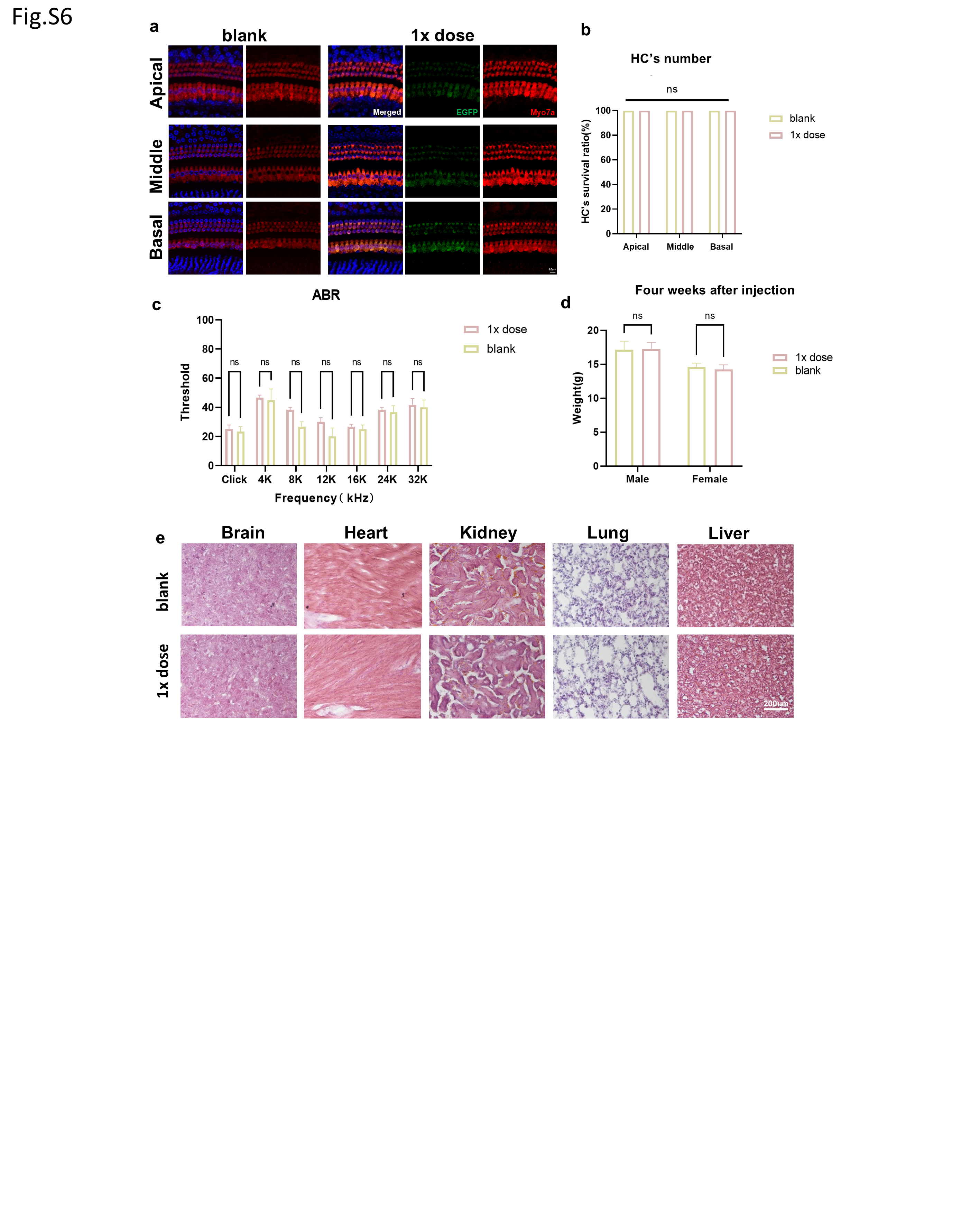
**

**Fig.S6 Single delivery of AAV-ie-Eh3-m*Atp6v1b2*-EGFP into the cochlea of wild-type（WT）mice has minimal adverse effects evaluated 4 weeks after injection.** a. No obvious morphological changes in hair cells (HCs) of cochlea was observed in AAV-ie-Eh3-m*Atp6v1b2*-EGFP injected mice. b. There was no difference in the number of HCs between the uninjected and injected groups (n=3 in each group). c. No significant ABR threshold difference was observed between the uninjected and injected groups (n=3 in each group). d. Compared to the uninjected WT mice, no significant difference in the body weight was found in mice injected with AAV-ie-Eh3-m*Atp6v1b2*-EGFP (n=6). e. Compared to the uninjected WT mice, HE staining of multiple organs showed no histological abnormality in mice injected with AAV-ie-Eh3-m*Atp6v1b2*-EGFP (n=3). Error bars indicated the standard deviation. The p-value was calculated by two-way ANOVA, and ns means no significant difference.

**Table S1. Full blood c**ount (FBC)

|  | blank | 1x dose |
| --- | --- | --- |
| Leukocyte count | 1.20x 10^9/L | 1.41x10^9/L |
| neutrophil count | 0.11 x 10^9/L | 0.16x10^9/L |
| Lymphocyte count | 1.03 x 10^9/L | 1.21x10^9/L |
| Monocyte count | 0.04 x 10^9/L | 0.03x10^9/L |
| Eosinophils count | 0.02 x 10^9/L | 0.01x10^9/L |
| Basophils count | 0.00 | 0.00 |
| Neutrophil percentage | 9.2% | 11.0 % |
| Lymphocyte percentage | 85.3% | 85.6 % |
| Monocyte percentage | 2.9% | 2.0% |
| eosinophils percentage | 2.0% | 1.3% |
| Basophils percentage | 0.6% | 0.1% |
| Red Blood Cell Count | 6.26x 10^12/L | 7.15x10^12/L |
| hemoglobin | 114 g/L | 128 g/L |
| Hematocrit | 35.1% | 41.4 % |
| Mean Corpuscular Volume | 56.0 fL | 57.9 fL |
| Mean Corpuscular Hemoglobin | 18.2pg | 17.9 pg |
| Mean Corpuscular Hemoglobin Concentration | 325 g/L | 308 g/L |
| Red Cell Distribution Width Coefficient of Variation | 14.6 % | 13.4 % |
| Red Cell Distribution Width Standard Deviation | 31.1 fL | 29.2 fL |
| platelet count | 306x10^9/L | 402x10^9/L |
| Mean Platelet Volume | 6.1fL | 6.9fL |
| Platelet Distribution Width | 15.9 | 15.8 |
| Thrombocytocrit | 0.186% | 0.276% |

Table S2. Blood biochemistry in mice

|  | blank | 1x dose |
| --- | --- | --- |
| Alanine Transaminase (U/L) | 85.539 | 45.491 |
| Aspartate Aminotransferase (U/L) | 179.680 | 128.834 |
| UREA (mmol/L) | 8.494 | 8.736 |
| Creatinine (μmol/L) | 14.262 | 10.785 |
| Cholesterol (mmol/L) | 1.475 | 1.791 |
| Albumin (g/L)  Total Bilirubin (TBIL) | 25.747  61.248 | 27.146  50.191 |

**P values** of Fig. 1

|  | Apex | Middle | Basal |
| --- | --- | --- | --- |
| P14 HC’s number | <0.0001 | <0.0001 | <0.0001 |

|  | Click | 4kHz | 8kHz | 12kHz | 16kHz | 24kHz | 32kHz |
| --- | --- | --- | --- | --- | --- | --- | --- |
| ABR | <0.0001 | <0.0001 | <0.0001 | <0.0001 | <0.0001 | <0.0001 | <0.0001 |

P values of Fig. 2

|  | Apex | Middle | Basal |
| --- | --- | --- | --- |
| P12 HC’s number | ＜0.0001 | ＜0.0001 | ＜0.0001 |

P values of Fig. 3

|  | Apex | Middle | Basal |
| --- | --- | --- | --- |
| P5 Single HC's lysosome volume | <0.0001 | <0.0001 | <0.0001 |

**P values of Fi**g. 4

|  | AAV2-CAG vs. AAV-ie-Eh3 | AAV8-CAG  vs. AAV-ie-Eh3 | AAV9-CAG  vs. AAV-ie-Eh3 | AAV-Anc80L65-CAG  vs. AAV-ie-Eh3 | AAV-ie-CAG  vs. AAV-ie-Eh3 |
| --- | --- | --- | --- | --- | --- |
| HC’s Specificity (%) | ＜0.0001 | ＜0.0001 | ＜0.0001 | ＜0.0001 | ＜0.0001 |

P values of Fig. 5

|  | *Atp6v1b2^fl/fl^*  vs. *Atp6v1b2^fl/fl^;Atoh1^cre/+^* | *Atp6v1b2^fl/fl^*  vs.  *Atp6v1b2^fl/fl^;Atoh1^Cre/+^*+AAV-ie-Eh3-m*Atp6v1b2*-EGFP |
| --- | --- | --- |
| Sectional Area of Lysosome | ＜0.0001 | 0.0168 |

P values of Fig. 6

|  | *Atp6v1b2^fl/fl^*  vs. *Atp6v1b2^fl/fl^;Atoh1^cre/+^* | *Atp6v1b2^fl/fl^*  vs.  *Atp6v1b2^fl/fl^;Atoh1^Cre/+^*+AAV-ie-Eh3-m*Atp6v1b2*-EGFP | *Atp6v1b2^fl/fl^;Atoh1^cre/+^*  vs.  *Atp6v1b2^fl/fl^;Atoh1^Cre/+^*+AAV-ie-Eh3-m*Atp6v1b2*-EGFP |
| --- | --- | --- | --- |
| Open Field Test  In border/center point | ＜0.0001 | 0.3593 | ＜0.0001 |

|  | *Atp6v1b2^fl/fl^*  vs. *Atp6v1b2^fl/fl^;Atoh1^cre/+^* | *Atp6v1b2^fl/fl^*  vs.  *Atp6v1b2^fl/fl^;Atoh1^Cre/+^*+AAV-ie-Eh3-m*Atp6v1b2*-EGFP | *Atp6v1b2^fl/fl^;Atoh1^cre/+^*  vs.  *Atp6v1b2^fl/fl^;Atoh1^Cre/+^*+AAV-ie-Eh3-m*Atp6v1b2*-EGFP |
| --- | --- | --- | --- |
| Open Field Test  In border/nose point | ＜0.0001 | 0.5886 | ＜0.0001 |

|  | *Atp6v1b2^fl/fl^*  vs. *Atp6v1b2^fl/fl^;Atoh1^cre/+^* | *Atp6v1b2^fl/fl^*  vs.  *Atp6v1b2^fl/fl^;Atoh1^Cre/+^*+AAV-ie-Eh3-m*Atp6v1b2*-EGFP | *Atp6v1b2^fl/fl^;Atoh1^cre/+^*  vs.  *Atp6v1b2^fl/fl^;Atoh1^Cre/+^*+AAV-ie-Eh3-m*Atp6v1b2*-EGFP |
| --- | --- | --- | --- |
| Open Field Test  In center/center point | ＜0.0001 | 0.3993 | ＜0.0001 |

|  | *Atp6v1b2^fl/fl^*  vs. *Atp6v1b2^fl/fl^;Atoh1^cre/+^* | *Atp6v1b2^fl/fl^*  vs.  *Atp6v1b2^fl/fl^;Atoh1^Cre/+^*+AAV-ie-Eh3-m*Atp6v1b2*-EGFP | *Atp6v1b2^fl/fl^;Atoh1^cre/+^*  vs.  *Atp6v1b2^fl/fl^;Atoh1^Cre/+^*+AAV-ie-Eh3-m*Atp6v1b2*-EGFP |
| --- | --- | --- | --- |
| Open Field Test  In center/nose point | ＜0.0001 | 0.5676 | ＜0.0001 |

|  | P14 | P30 | P45 | P60 | P90 |
| --- | --- | --- | --- | --- | --- |
| Rotarod Test | ＜0.0001 | ＜0.0001 | ＜0.0001 | ＜0.0001 | ＜0.0001 |

P values of Fig. S3

|  | Apex | Middle | Basal |
| --- | --- | --- | --- |
| P0 Single HC's lysosome volume | <0.0001 | <0.0001 | <0.0001 |

|  | Apex | Middle | Basal |
| --- | --- | --- | --- |
| P3 Single HC's lysosome volume | <0.0001 | <0.0001 | <0.0001 |

|  | Apex | Middle | Basal |
| --- | --- | --- | --- |
| P7 Single HC's lysosome volume | <0.0001 | <0.0001 | <0.0001 |

|  | Apex | Middle | Basal |
| --- | --- | --- | --- |
| P9 Single HC's lysosome volume | <0.0001 | <0.0001 | <0.0001 |

|  | Apex | Middle | Basal |
| --- | --- | --- | --- |
| P12 Single HC's lysosome volume | <0.0001 | <0.0001 | <0.0001 |

P values of Fig. S6

|  | Apex | Middle | Basal |
| --- | --- | --- | --- |
| HC’s number | 1 | 1 | 1 |

|  | Click | 4kHz | 8kHz | 12kHz | 16kHz | 24kHz | 32kHz |
| --- | --- | --- | --- | --- | --- | --- | --- |
| ABR | 1 | 1 | 0.2695 | 0.4472 | 1 | 1 | 1 |

|  | Male | Female |
| --- | --- | --- |
| Four weeks after injection | 0.9496 | 0.6728 |
